# Novel serum biomarker for powerfully predicting pediatric simple febrile seizures

**DOI:** 10.3389/fped.2025.1646261

**Published:** 2025-08-14

**Authors:** Jinzhi Mei, Chuanze Hu, Fang Sheng, Zhiyu Wang, Wangxiong Hu

**Affiliations:** ^1^ Department of Pediatrics, Jinhua Maternal and Child Health Care Hospital, Jinhua, Zhejiang, China; ^2^ Cancer Institute (Key Laboratory of Cancer Prevention and Intervention, China National Ministry of Education), The Second Affiliated Hospital, Zhejiang University School of Medicine, Hangzhou, Zhejiang, China

**Keywords:** febrile seizures, IL-6, IFN-α, vitamin D, ROC curve

## Abstract

**Objective:**

Current model or biomarkers are far from satisfactory to predict pediatric simple febrile seizures (SFS). This study aimed to explore novel serum biomarkers in children with SFS and analyze their clinical significance.

**Methods:**

A total of 57 SFS children admitted to the pediatric ward of Jinhua Maternal and Child Health Hospital from June 2022 to October 2024 were enrolled as the observation group, 61 sepsis patients and 45 healthy children from the pediatric health clinic during the same period were included as the control groups. Serum levels of 25-hydroxyvitamin D and diverse cytokines were measured using enzyme-linked immunosorbent assay (ELISA). Univariate logistic regression was used to identify the predictive ability of risk factors for SFS.

**Results:**

The SFS group exhibited significantly lower levels of IL-17 (SFS: 5 vs. Health: 12 and Sepsis: 16) compared to the control groups. Conversely, the SFS group showed significantly higher levels of pro-inflammatory cytokines (IFN-α: 22 vs. 4 and 5; and IL-10: 12 vs. 10 and 9) than the control groups. ROC curve revealed that during SFS episodes, IFN-α can powerfully in discriminating SFS and health cohorts.

**Conclusion:**

Serum level of IFN-α hold significant predictive value for SFS occurrence.

## Introduction

Febrile seizures (FS) are the most common convulsive disorder in childhood, usually associated with a fever of ≥38°C and an incidence of 2%–11% (1, 2). The highest incidence of FS is at 18 months and prevalence peaking in children aged 6 months to 5 years without central nervous system infection nervous system infections or other etiologies of seizures, and with no prior history of febrile seizures (3). According to the 2011 diagnostic criteria established by the American Academy of Pediatrics (AAP) (4), simple febrile seizures (SFS) are defined as generalized tonic-clonic convulsion that spontaneously resolve within 15 min, with return to an alert mental status, and do not recur within a 24 h period, in a patient aged 6–60 months, associated with documented fever higher than 38°C, in the absence of a pre-existing metabolic disorder, neurological abnormality or previous seizure.

Despite the majority of affected children have a good prognosis, they carry a risk of subsequent epilepsy, impacting children's cognition and increasing socioeconomic burdens (5). The exact causes of SFS remain unknown, although some studies indicate a possible association with environmental and genetic factors (6, 7). Therefore, understanding the risk factors for SFS is crucial for implementing early interventions to mitigate long-term complications (8). Previously much efforts have been made to predict FS but demonstrated limited predictive ability, with AUC values <0.8, including serum brain-derived neurotrophic factor (AUC = 0.723) (9), neurotrophin-3 (AUC = 0.678) (10), hypomagnesemia (AUC = 0.731) (11), neutrophil to lymphocyte ratio (NLR, AUC = 0.768) and mean platelet volume (MPV)/platelet count (PLT) ratio (MPR, AUC = 0.689) (12), neutrophil lymphocyte platelet ratio (NLPR, AUC = 0.774) (13). Therefore, our study aims to develop SFS predictive markers that can significantly improve predictive ability and holds practical value in clinic.

Research has demonstrated that FS and convulsions induce neuronal damage mediated by inflammatory and immune responses (14). However, limited studies report the relationship between serum cytokine profiles and clinical manifestations in SFS patients. It is thus tempting to believe that cytokines that play important roles in immune microenvironment may serve as promising biomarkers in SFS prediction.

In this study, we collected serum cytokines and demographic data in retrospective cohorts of children with SFS, sepsis patients and healthy controls. Next, we investigated sensitivity and specificity of individual variables, aiming to establish a robust risk prediction models and innovative strategies for clinical diagnosis and early prevention.

## Materials and methods

### Study population

This retrospective study was designed to analyze SFS cases admitted to the department of pediatrics at Jinhua maternal and child health care hospital between June 2022 and October 2024.Inclusion criteria encompassed: (1) Children meeting the diagnostic criteria for SFS; (2) Age at 2–5 years old; (3) No recent supplementation with vitamin D-containing medications; and (4) First episode of seizure. Exclusion criteria comprised: (1) central nervous system infections, fever-sensitive epilepsy, immune-mediated encephalitis, inherited metabolic disorders, or similar conditions; (2) Electrolyte imbalances, hypoglycemia, or related abnormalities; (3) Intracranial tumors; (4) Vitamin D deficiency cases secondary to malnutrition; and (5) There have been episodes of seizure in the past. Finally, 57 pediatric patients with SFS were admitted in this study, 61 sepsis patients (who experienced fever above 38°C but did not develop seizures) and 45 age-matched healthy children undergoing routine physical examinations at the pediatric healthcare outpatient clinic during the same period were included as the control groups. The research protocol was approved by the Medical Ethics Committee of Jinhua maternal and child health care hospital. Written informed consent forms were obtained from all legal guardians of the participants prior to enrollment.

### Data collection

From the medical records, we gathered clinical details about children (including age, sex, and family history of FS or epilepsy), disease characteristics (including duration of the first episode, highest temperature before the first episode, and number of seizures episodes prior to the visit), and serum examination information (25-Hydroxyvitamin D (OHD25), interleukin 1β (IL-1β), IL-2, IL-4 IL-5, IL-6, IL-8, IL-10, IL-12, tumor necrosis factor α (TNF-α), interferon α (INF-α), and INF-γ). All blood tests were conducted on the consultation day. Briefly, 3 ml of venous blood from admitted SFS within one hour of admission, and serum OHD25 (Zhejiang Diseth Diagnostics Co., LTD., batch No. K2407023) and cytokine (cytokine detection kit, Qingdao Kaisirui Biotechnology Co., Ltd., Lot No. 240601) levels were tested by enzyme-linked immunosorbent assay (ELISA) in strict accordance with the kit instructions to complete the specimen test.

### Statistical analysis

Data were presented as counts (percentages) for categorical variables such as sex, and group-wise comparisons were conducted using the chi-squared test. Data were described as mean and standard deviation (SD) for continuous variables and compared using the *t* test or rank sum test (Mann–Whitney test) for non-normally distributed measurement data. *P* < 0.05 was used as the criterion for statistically significant differences. Risk factors for occurrence of SFS analyze using a univariate logistic regression model. The area under the curve (AUC) was performed by *pROC* package in R. The optimal cutoff values for the biomarkers were determined based on the Youden index maximization principle. All data analysis was performed by R (V. 4.2.3) unless otherwise stated.

## Results

A total of 57 children (34 males and 23 females, 1.48:1) with SFS were included in this study, with a median age of 3 years, while the health and sepsis control groups included 45 cases (23 males and 22 females, 1.05:1) and 61 cases (30 males and 31 females, 0.97:1) with a median age of 4- and 5- years, respectively. All participants were aged 2–9 years. There was no statistically significant difference in sex between the three groups (*P* > 0.05, Table 1), while the sepsis group had an older age than the SFS and health groups.

**Table 1:** Comparison of general data between the SFS and control groups.

Characteristic	Overall	Health	Sepsis	SFS	*p*
*n*	163	45	61	57	
Age [mean (SD)]	4.27 (2.25)	3.61 (0.97)	5.72 (2.89)	3.24 (1.10)	<0.001
Sex = male (%)	87 (53.4)	23 (51.1)	30 (49.2)	34 (59.6)	0.49

**Figure 1:**
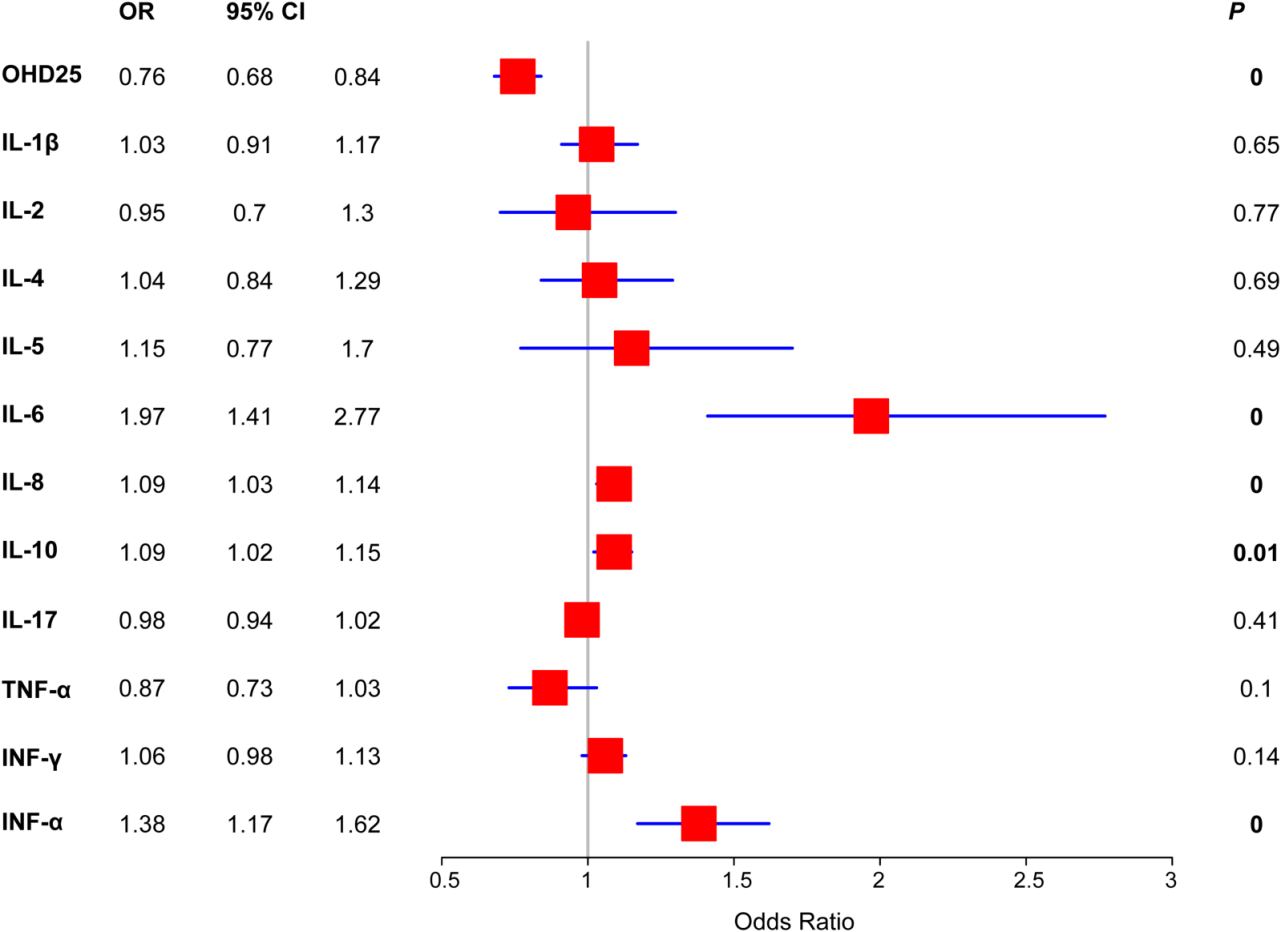
Forest plot of the SFS risk of serum markers detected in this study. Binary logistic regression was used to determine the risk of each serum markers.

As for the serum markers, we found that the SFS group had significantly lower cytokine IL-17 (5 vs. 12 and 16, *P* = 0.003) than the control groups (Table 2). In contrast, inflammatory cytokines such as IL-10 (12 vs. 10 and 9) and INF-α (22 vs. 4 and 5) were significantly up-regulated in the SFS group. To further understand the correlation between risk factors and SFS occurrence, we first performed univariate logistic regression based on the SFS and health groups. The results showed that OHD25 was associated with decreased risk of SFS (OR = 0.76, 95% CI: 0.68–0.84, *P* < 0.001), the higher OHD25 conferred a lower the risk of SFS occurrence. On the contrary, the high levels of IL-6 (OR = 1.97, 95% CI: 1.41–2.77, *P* < 0.001), IL-8 (OR = 1.09, 95% CI: 1.03–1.14, *P* < 0.001), IL-10 (OR = 1.09, 95% CI: 1.02–1.15, *P* = 0.01) and IFN-α (OR = 1.38, 95% CI: 1.17–1.62, *P* < 0.001) were associated with increased risk of SFS occurrence (Figure 1).

**Table 2:** Comparison of serum OHD25 (ng/ml) and cytokines (pg/ml) levels between SFS and two control groups.

Characteristic	Overall	Group			*p*-value^b^
	*N* = 163^a^	SFS	Health	Sepsis	
		*N* = 57^a^	*N* = 45^a^	*N* = 61^a^	
OHD25	30 (26, 38)	29 (27, 32)	39 (37, 42)	24 (19, 33)	**<0** **.** **001**
IL1B	3.35 (1.47, 5.42)	2.38 (1.47, 4.50)	3.35 (1.08, 5.42)	3.65 (2.13, 5.96)	0.175
IL.2	1.68 (0.94, 2.57)	1.60 (0.70, 2.41)	1.50 (1.02, 2.49)	1.85 (1.18, 2.98)	0.221
IL.4	1.61 (0.75, 2.80)	1.71 (1.02, 2.37)	1.60 (0.58, 2.80)	1.65 (0.56, 3.07)	0.823
IL.5	0.78 (0.47, 1.26)	0.91 (0.49, 1.26)	0.78 (0.50, 1.26)	0.75 (0.40, 1.20)	0.675
IL.6	12 (4, 37)	15 (7, 44)	4 (3, 5)	32 (15, 62)	**<0** **.** **001**
IL.8	14 (9, 26)	16 (10, 39)	10 (4, 13)	23 (13, 31)	**<0** **.** **001**
IL.10	10 (6, 15)	12 (7, 26)	10 (6, 12)	9 (7, 14)	**0** **.** **015**
IL.17	10 (2, 17)	5 (2, 13)	12 (4, 15)	16 (6, 27)	**0** **.** **003**
TNF-α	2.07 (1.13, 3.97)	1.80 (1.19, 3.28)	2.27 (0.94, 4.07)	2.77 (1.49, 4.19)	0.258
INF-r	4.1 (2.2, 7.4)	3.7 (1.6, 8.6)	3.6 (2.3, 5.4)	5.0 (2.8, 7.8)	0.061
INF-α	6 (3, 17)	22 (10, 68)	4 (3, 6)	5 (3, 9)	**<0** **.** **001**

Bold *p*-values represent the statistically significant results.

^a^ Median (Q1, Q3).

^b^ Kruskal–Wallis rank sum test.

**Figure 2:**
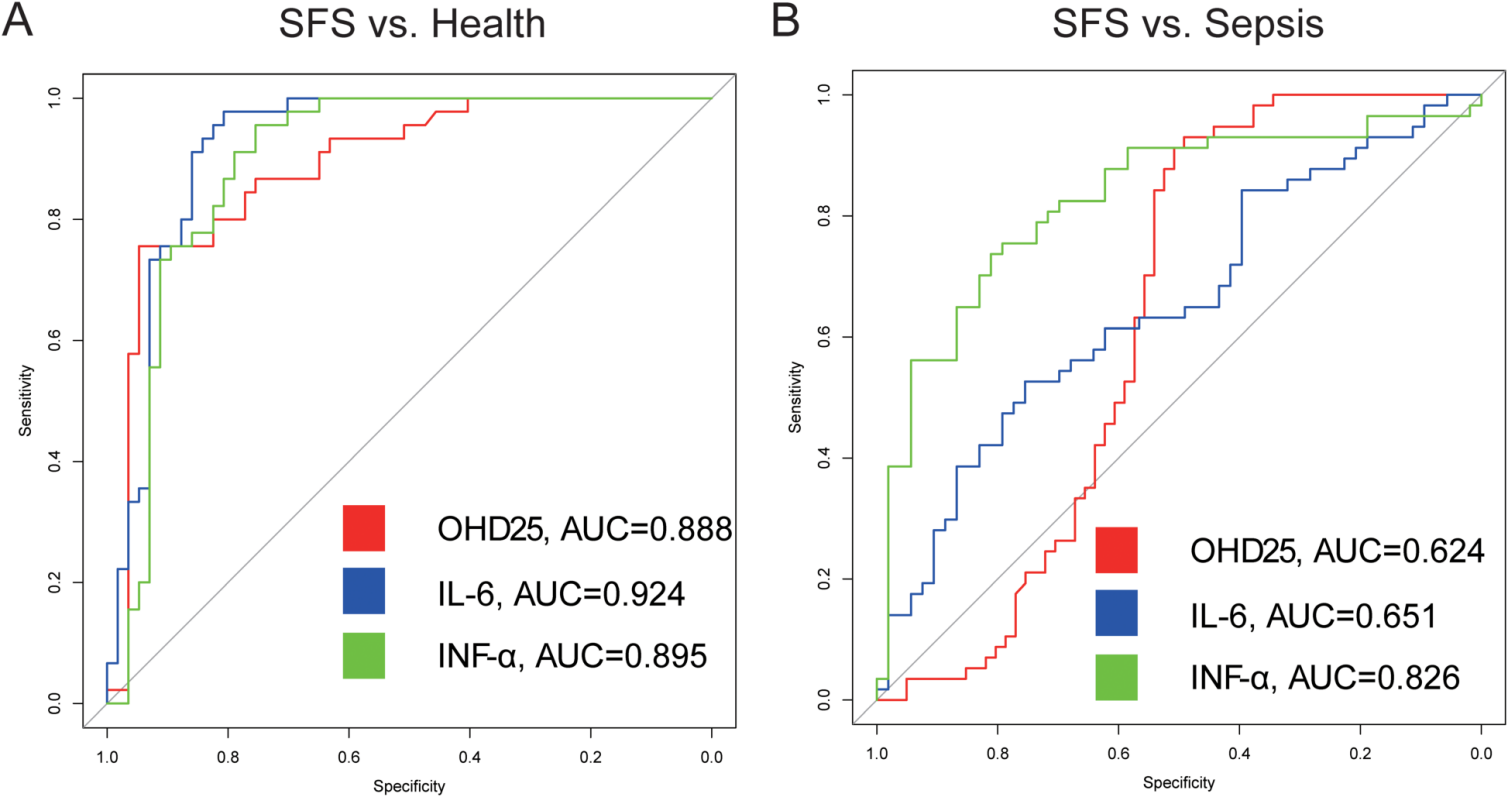
SFS ROC curve. **(A)** Receiver operating characteristics of OHD25, IL-6 and INF-α in discriminating SFS from health group. **(B)** Receiver operating characteristics of OHD25, IL-6 and INF-α in discriminating SFS from sepsis group.

To further identify the novel risk factors in predicting SFS occurrence, we performed ROC curve analysis and we found that three biomarkers showed excellent prediction power in determining SFS occurrence. The OHD25 [optimal cutoff value = 36.335 ng/ml, sensitivity = 94.7%, specificity = 75.6%; AUC = 0.888, 95% confidence interval (95% CI) = 0.821–0.954], IL-6 (optimal cutoff value = 6.425 pg/ml, sensitivity = 80.7%, specificity = 97.8%, AUC = 0.924, 95% CI = 0.869–0.979) and IFN-α (optimal cutoff value = 9.375 pg/ml, sensitivity = 75.4%, specificity = 95.6%, AUC = 0.895, 95% CI = 0.829–0.962) demonstrated high predictive value for SFS, with IL-6 exhibiting the highest discriminative power (AUC = 0.924, Figure 2A).

Then, to rule out the possibility of nonspecific increased level of cytokines because of fever, we also compared the discriminative power between the SFS and the sepsis groups. Results showed that IFN-α (AUC = 0.826) also demonstrated high predictive value for SFS, which was consistent with the above comparison results (Figure 2B).

## Discussion

SFS represent the most common form of seizures in children (15).The identification of novel biomarkers during SFS episodes holds significant potential for predicting SFS occurrence and enabling early preventive interventions to avoid abuse treatment and physical examination (16). Our study reveals elevated serum levels of pro-inflammatory cytokines (IL-6, IL-8, IL-10, and IFN-α) in SFS patients, indicating their contributory role in FS initiation. In addition, the robust performance of mono indicator IFN-α (AUC > 0.8) in predicting SFS compared to both sepsis and health groups is superior to other published models and markers. Excessive pro-inflammatory cytokine release may induce blood-brain barrier (BBB) dysfunction, neuronal injury, and precipitating seizures. Thus, dysregulation of the pro-inflammatory cytokine equilibrium may constitute a pivotal mechanism underlying FS development.

Huang et al. (17) found that seizure episodes in rats are associated with significantly elevated levels of inflammatory cytokines IL-6 and TNF-α. Increased IL-6 expression may activate NMDA receptors, leading to impaired cerebral autoregulation and hippocampal neuronal damage. IL-8, a pro-inflammatory cytokine and neutrophil-activating peptide, is primarily secreted by monocyte-derived macrophages, astrocytes, and microglia. It plays a critical role in post-injury neuronal repair by promoting neurite outgrowth and stimulating neurotrophic factor production. Clinical evidence indicates that IL-8 exacerbates seizure activity, with its elevation correlating positively with seizure severity (18). By altering the pro-convulsive microenvironment, IL-8 may further potentiate FS development. Consistent with these findings, our study demonstrates marked upregulation of IL-6 and IL8 during SFS episodes, suggesting their promotive role in SFS development. Notably, early anti-inflammatory therapy could be considered for children with pronounced inflammatory responses to mitigate SFS progression to epilepsy.

Another interesting finding in this study is the markedly decreased OHD25 in the fever groups (SFS and sepsis) than the health control group. Bhat et al. (19) found that 43.5% of children with SFS exhibit insufficient serum OHD25 levels, while 30.85% demonstrates deficiency and 25.56% maintain normal levels, which was consistent with our observation in this study. Additionally, injected subcutaneously to NMRI mice with vitamin D supports the direct anticonvulsant role of vitamin D in the brain (20). Therefore, routine monitoring of serum OHD25 is recommended for children over 2 years old, not only to support growth but also to mitigate SFS risk, as vitamin D deficiency constitutes an independent risk factor for SFS.

Taken together, IFN-α emerge as a novel promising biomarker for SFS diagnosis. Early intervention in SFS patients could improve prognosis but warrant validation through large-scale multicentric trials.

## Data Availability

The original contributions presented in the study are included in the article/Supplementary Material, further inquiries can be directed to the corresponding authors.
